# Responses of Soybean Genes in the Substituted Segments of Segment Substitution Lines Following a *Xanthomonas* Infection

**DOI:** 10.3389/fpls.2020.00972

**Published:** 2020-07-02

**Authors:** Jianan Zou, Zhanguo Zhang, Siyang Yu, Qinglin Kang, Yan Shi, Jinhui Wang, Rongsheng Zhu, Chao Ma, Lin Chen, Jieqi Wang, Jianyi Li, Qingying Li, Xueying Liu, Jingyi Zhu, Xiaoxia Wu, Zhenbang Hu, Zhaoming Qi, Chunyan Liu, Qingshan Chen, Dawei Xin

**Affiliations:** College of Agriculture, Key Laboratory of Soybean Biology in Chinese Ministry of Education, Northeast Agricultural University, College of Science, Harbin, China

**Keywords:** soybean, *Xanthomonas*, HrpG, RNA-seq, chromosome substituted segment

## Abstract

Bacterial blight, which is one of the most common soybean diseases, is responsible for considerable yield losses. In this study, a novel *Xanthomonas vasicola* strain was isolated from the leaves of soybean plants infected with bacterial blight under field conditions. Sequencing the *X. vasicola* genome revealed type-III effector-coding genes. Moreover, the *hrpG* deletion mutant was constructed. To identify the soybean genes responsive to HrpG, two chromosome segment substitution lines (CSSLs) carrying the wild soybean genome, but with opposite phenotypes following *Xanthomonas* inoculations, were used to analyze gene expression networks based on RNA sequencing at three time points after inoculations with wild-type *Xanthomonas* or the *hrpG* deletion mutant. To further identify the hub genes underlying soybean responses to HrpG, the genes located on the substituted chromosome segments were examined. Finally, a combined analysis with the QTLs for resistance to *Xanthomonas* identified 35 hub genes in the substituted chromosomal segments that may help regulate soybean responses to *Xanthomonas* and HrpG. Furthermore, two candidate genes in the CSSLs might play pivotal roles in response to *Xanthomonas.*

## Introduction

Soybean [*Glycine max* (L.) Merr.] seeds are rich in proteins and the largest contributors to the worldwide supply of the edible oil and protein used in food, feed, and industrial products (Lam et al., 2010; Whitham et al., 2016; Gao et al., 2018). In China, Heilongjiang is the main soybean-producing province, responsible for more than 30% of the soybean produced in the country (Tian et al., 2016). However, soybean production in Heilongjiang has been limited by soybean bacterial leaf pustule (BLP) caused by *Xanthomonas*, which is one of the top-10 plant pathogenic bacteria infecting important crops, including soybean, rice, pepper, and citrus (Mansfield et al., 2012). The virulence and pathogenicity of *Xanthomonas* have been elucidated in genomic studies that identified many effectors delivered into hosts *via* a type-III secretion system (T3SS) (Roach et al., 2019).

The pathogenicity of *Xanthomonas* relies on type-III effectors secreted by the T3SS, which is encoded by the chromosomal *hrp* (hypersensitive response and pathogenicity) gene cluster (Schulte and Bonas, 1992; Wengelnik et al., 1999). Previous studies revealed that HrpG is a pivotal regulator underlying T3SS activity (Rashid et al., 2016; Hu et al., 2018). A mutated HrpG results in the expression of *hrp* genes even under non-*hrp*-inducing conditions in *Xanthomonas campestris* pv. *vesicatoria* (Wengelnik et al., 1999; Moss et al., 2007). Thirty HrpG-induced genes and five HrpG-repressed genes have been identified in *X. campestris* pv. *vesicatoria* (Büttner and Bonas, 2010; Tampakaki et al., 2010). Additionally, genome-wide microarray analyses confirmed that *hrpG in Xanthomonas axonopodis* pv. *citri* helps regulate multiple physiological functions, including biofilm formation (Guo et al., 2011). During infections of rice, the phosphorylation of HrpG in *Xanthomonas oryzae* pv. *oryzae* is essential for the induction of *hrp* expression, although the plant signal that regulates this phosphorylation is unknown (Li et al., 2014).

Several genes and quantitative trait loci (QTLs) for BLP resistance have been identified *via* genetic mapping, including the mapped recessive gene (*rxp*) on chromosome 17 and QTLs located in the region between Satt372 and Satt486 (Palmer et al., 1992; Narvel et al., 2001; Van et al., 2004; Choi et al., 2007). The *rxp* gene has yet to be isolated, and the defense mechanism underlying soybean resistance to BLP remains unclear (Kim et al., 2010).

Chromosome segment substitution lines (CSSLs) are useful and valuable materials for gene mapping. Many critical genes underlying important traits have been identified by localizing QTLs in the CSSLs of rice, maize and tomato (Swanson-Wagner et al., 2012; Qiao et al., 2016; Capel, 2017; Xie et al., 2019). In soybean, near-isogenic lines have been applied to identify target genes, proving to be more useful than other populations such as recombinant inbred lines (RILs) (Keurentjes et al., 2007; Xin et al., 2016). An earlier comprehensive transcriptome analysis provided new insights into developmentally and environmentally induced changes in gene expression (Kim et al., 2011). Moreover, combining QTL analyses with transcriptomics-based investigations represents a powerful strategy for predicting the roles and interactions of individual genes and for elucidating the mechanisms mediating soybean–bacterial pathogen interactions.

In this study, the *hrpG* deletion mutant was constructed based on the genome sequence of *Xanthomonas* NEAU001 (XvNEAU001WT), which was isolated from a soybean field in Heilongjiang. Additionally, two CSSLs (containing different wild soybean genomic segments) exhibiting the opposite phenotypes after being inoculated with XvNEAU001WT underwent a transcriptome analysis. The 81 obtained RNA sequencing (RNA-seq) datasets revealed extensive gene expression differences between the two CSSLs in response to the *hrpG* mutant and the parental strain (XvNEAU001WT). Moreover, the diversity in the gene expression patterns induced by the *hrpG* mutant and the parental strain was determined. Finally, the candidate genes involved in the T3SS pathway were analyzed. A genome-wide linkage of differentially expressed genes (DEGs), QTLs (disease spots), and selection sweeps of chromosomes 08 and 11 implied that DEGs located on the substituted segments of wild soybean might be the key genes regulating soybean resistance to *Xanthomonas*. Our results provide new insights into the soybean–*Xanthomonas* interaction and reveal candidate genes involved in the signaling pathway induced by HrpG and other unidentified effectors secreted by T3SS.

## Results

### Isolation of *Xanthomonas* and Determination of Pathogenicity

**Figure 1:**
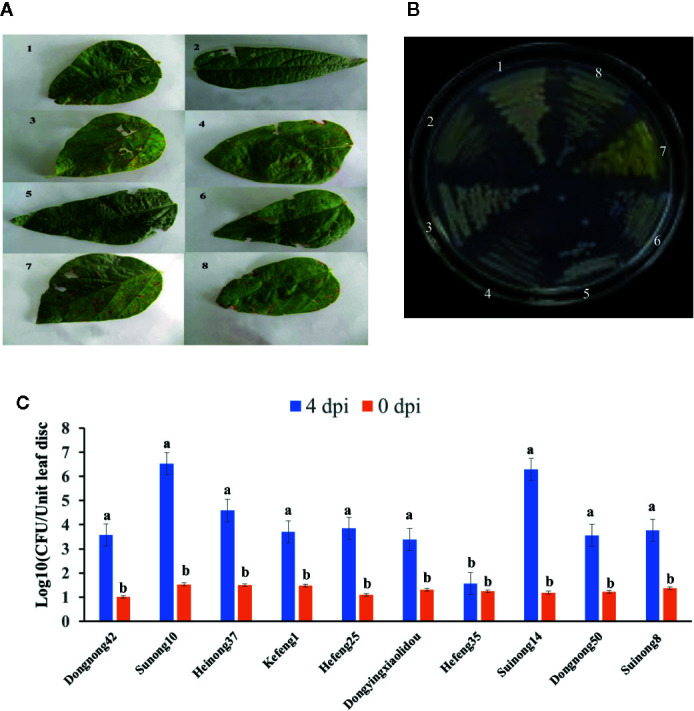
Isolation and pathogenicity analysis of XvNEAU001WT. **(A)** BLP leafs isolated for the soybean field. **(B)** candidate bacterial pathogen cones isolated from the leaf. **(C)** Phenotype analysis of soybean germplasm after 4 days inoculated with XvNEAU001WT. Leaves 1–6 were isolated from the soybean field of Harbin, Leaves 7 and 8 were isolated from the soybean field of Jiamusi. The bar and error bars is the mean and standard deviation. Bars with different letters are significantly different at p <0.05 as analyzed by Duncan’s multiple comparison test. The circle marked region was isolated sed to perform RNA-sequencing. The days to measurement of the bacterial growth were four days after inoculation. Three trifoliate were used to calculate the number. The biological analysis on soybean germplasm was conducted for three times.

Soybean leaves with pustules were isolated from plants grown in fields of Harbin and Jiamusi after a heavy rainfall (**Figure 1A**). A pathogenic *Xanthomonas* clone was isolated. More than 2 days were required for this clone to grow at 28°C, after which the 16S rDNA from this clone was amplified and sequenced (**Table 1**). A BLAST analysis of the NCBI database (https://blast.ncbi.nlm.nih.gov/) with the 16S rDNA as a query indicated that clone #7 was similar to *Xanthomonas vasicola* LMG 736(T), whereas the other clones most closely matched *Pseudomonas* (**Table 1**). The phenotype of clone #7 is also different from other clones (**Figure 1B**). Thus, the isolated *Xanthomonas* was named *X. vasicola* NEAU001 (XvNEAU001WT). To determine the antibiotic of XvNEAU001WT, we tested its resistance to antibiotics. The results indicated that XvNEAU001WT is resistant to 50 µg ml^−1^ spectinomycin, but not to 100 µg ml^−1^ spectinomycin or rifampicin, kanamycin, chloramphenicol, gentamicin, and tetracycline.

**Table 1:** The closest match of bacterial 16Sr DNA.

No.	16s length (bp)	Similarity bacteria	Similarity%	Accession
*Psgneau1*	1,502	*Pseudomonas congelans DSM 1439(T)*	99.67	AJ492828
*Psgneau2*	1,497	*Pseudomonas ficuserectae JCM 2400(T)*	99.86	AB021378
*Psgneau3*	1,499	*Pseudomonas congelans DSM 1439(T)*	99.67	AJ492828
*Psgneau4*	1,500	*Pseudomonas ficuserectae JCM 2400(T)*	99.93	AB021378
*Psgneau5*	1,500	*Pseudomonas ficuserectae JCM 2400(T)*	99.72	AB021378
*Psgneau6*	1,501	*Pseudomonas ficuserectae JCM 2400(T)*	99.86	AB021378
*Xaneau7*	1,508	*Xanthomonas vasicola LMG 736(T)*	100.00	Y10755
*Psgneau 8*	1,500	*Pseudomonas ficuserectae JCM 2400(T)*	99.93	AB021378

The inoculation of various soybean germplasm with XvNEAU001WT indicated that the virulence of this bacterial isolate varies depending on the germplasm. For example, XvNEAU001WT was most virulent on Suinong10 and Suinong14, whereas it was most weakly virulent on Hefeng35 (**Figure 1C**).

### Genome Sequencing and Construction of the *hrpG* Deletion Mutant

The sequencing of the XvNEAU001WT genome revealed basic features, including 5, 221, 943 bp assembled in a single chromosome (GenBank: PRJNA564183), with no apparent autonomous plasmids, and an average GC content of 65.17%. Additionally, most of the identified genomic sequence encodes proteins, with 4,542 open reading frames predicted to encode polypeptides belonging to 3,995 protein families. Moreover, 547 unclustered genes were detected, as were conserved type-III effector genes, including *hrpG* and *hrpX* (**Dataset S1**).

**Figure 2:**
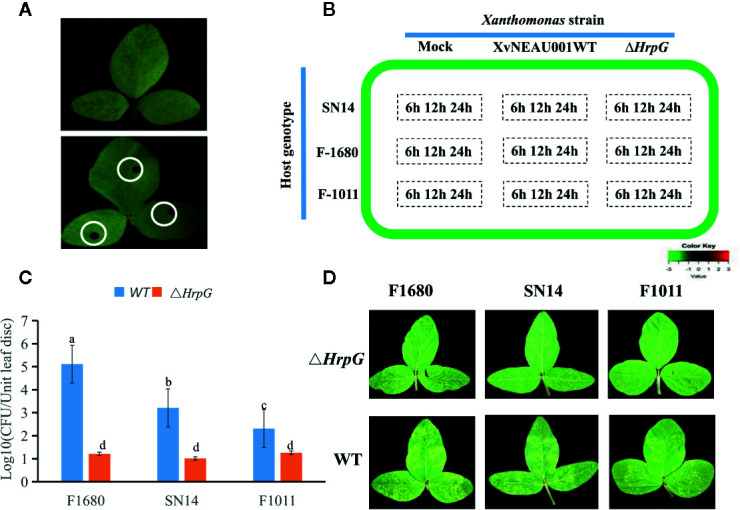
Phenotype analysis of F1011 and F1680. **(A)** phenotype of soybean first trifoliolate leaves after 4 days inoculated with *Xanthomonas*, bacteria number of leaf lesion (about 0.2 cm^2^) was counted on King’s medium B and used for phenotype evaluation. **(B)** RNA-seq treatment and sampling design. **(C, D)** Virulence analysis and phenotype of XvNEAU001WT and XvNEAU001ΔHrpG mutant on F1680, F1011 and their recurrent parent Suinong 14 (SN14). The bar and error bars is the mean and standard deviation. Bars with different letters are significantly different at p < 0.05 as analyzed by Duncan’s multiple comparison test. The days to measurement of the bacterial growth was four days after inoculation. Three trifoliate were used to calculate the number. The biological analysis on soybean germplasm was conducted for three times. Δ*HrpG* represents XvNEAU001Δ*HrpG*, WT represents XvNEAU001.

To determine the effects of HrpG on the pathogenicity of the bacterial pathogen on soybean, we developed the *hrpG* deletion mutant (XvNEAU001Δ*hrpG*). Compared with the wild-type strain, XvNEAU001Δ*hrpG* was significantly less virulent (**Figure 2C**). We also inoculated plants in the CSSL and RIL populations with XvNEAU001WT and the derived XvNEAU001Δ*hrpG*. Two CSSLs with the opposite phenotypic responses to the XvNEAU001WT inoculation were identified (**Figure 2D**). These results suggested that HrpG is important for the virulence of XvNAEU001.

### Soybean RNA-Seq Signatures Influenced by HrpG

To identify soybean genes controlled by virulence factors caused by HrpG in XvNEAU001WT, we profiled the leaf RNA-seq under 27 conditions (81 samples in total; **Figures 2A, B**, **Dataset S1**). Regarding the leaf transcriptome analysis, two CSSLs, F1011 and F1680, and their female parent Suinong14, were inoculated with wild-type XvNEAU001WT and XvNEAU001Δ*hrpG*, respectively. After inoculation infected leaves were collected at 6, 12, and 24 h post-inoculation (hpi). A total of 39, 921, 664 clean reads for each line were mapped to the Williams 82 reference genome (https://phytozome.jgi.doe.gov/pz/portal.html). The mapping rate ranged from 86.1 to 89.29%.

### A Negative or Positive Correlated Gene Co-Expression Module Related to the Resistance to XvNEAU001WT and HrpG Was Identified in Wild Soybean Substituted Fragments

**Figure 3:**
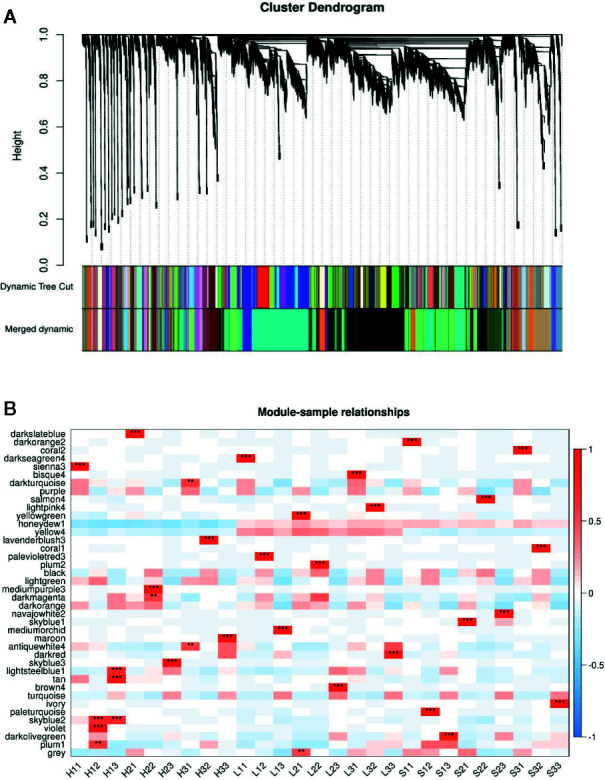
WGCNA based on the gene expression matrix between Sunong14, F1011 and F1680. **(A)** Hierarchical cluster tree showing co-expression modules identified by WGCNA. Each leaf in the tree represents one gene. The major tree branches constitute 40 modules labeled with different colors. **(B)** Module–sample association. Each row corresponds to a module labeled with a color as in **(A)** Modules are distinguished by different colors which were arbitrarily assigned by the WGCNA package. Each column corresponds to a tissue type as indicated. The color of each cell at the row–column intersection indicates the correlation coefficient (R) between the module and the tissue type. *Significance at P<0.05; **Significance at P<0.01.

To compare the overall gene expression patterns between F1680 and F1011, the transcriptomic data underwent a K-means clustering analysis, which enabled the examination of the gene expression changes over time in leaves inoculated with XvNEAU001WT or the *hrpG* deletion mutant. A total of 39,783 detected genes were grouped into 20 gene co-expression clusters (C1–C20). Finally, 4,624 distinct DEGs were identified between F1680 and F1011. The genes within each cluster exhibited a similar expression pattern, whereas the genes in different clusters had distinct expression patterns. The expression levels of genes in four clusters (C4, C5, C6, and C8) were obviously different between F1680 and F1011 in response to XvNEAU001WT and the *hrpG* mutant. The genes in the remaining 16 clusters were generally similarly expressed, with normal variation patterns, but some differences were detected between F1680 and F1011 following the inoculations with XvNEAU001WT and XvNEAU001Δ*hrpG* (**Figure 3**, **Dataset S2**). Furthermore, XvNEAU001Δ*hrpG* induced significant differences in the expression of the cluster C4 genes between F1011 and F1680 at 6, 12, and 24 hpi. A total of 1,897 genes in clusters C4, C5, C6, and C8 were differentially expressed between F1011 and F1680, representing approximately 41% of the total number of DEGs.

A gene ontology (GO) analysis was conducted to functionally characterize genes, with a particular focus on clusters C4, C5, C6, and C8 (**Table S3**). The cluster C5 genes were enriched with the following GO terms: intracellular organelle (42 genes), organelle (42 genes), intracellular membrane-bounded organelle (32 genes), and membrane-bounded organelle (32 genes). Additionally, cellular process and metabolic process were the main processes associated with the C5 genes. The genes in cluster C6 were mainly annotated with the following GO terms: binding (164 genes), cellular process (118 genes), metabolic process (142 genes), cellular metabolic process (106 genes), primary metabolic process (118 genes), and macromolecule metabolic process (108 genes). Most of the cluster C8 genes were annotated with the cellular process (36 genes) and metabolic process (48 genes) GO terms. In cluster C4, most of the genes were annotated with the metabolic process (208 genes), catalytic activity (201 genes), oxidoreductase activity (67 genes), and oxidation reduction (59 genes) GO terms. Genes encoding a PR1 homologous protein and a WRKY family transcription factor were also detected. In plants, *PR1* and *WRKY* genes are crucial for regulating pathogen resistance (Xu et al., 2006; Lincoln et al., 2018; Wenke, 2019), indicating that they may be key genes contributing to the soybean resistance to *Xanthomonas*.

### Identification of Positively Correlated Genes in the Substituted Segments Responsive to *Xanthomonas* and HrpG *via* the Gene Co-Expression Module

**Figure 4:**
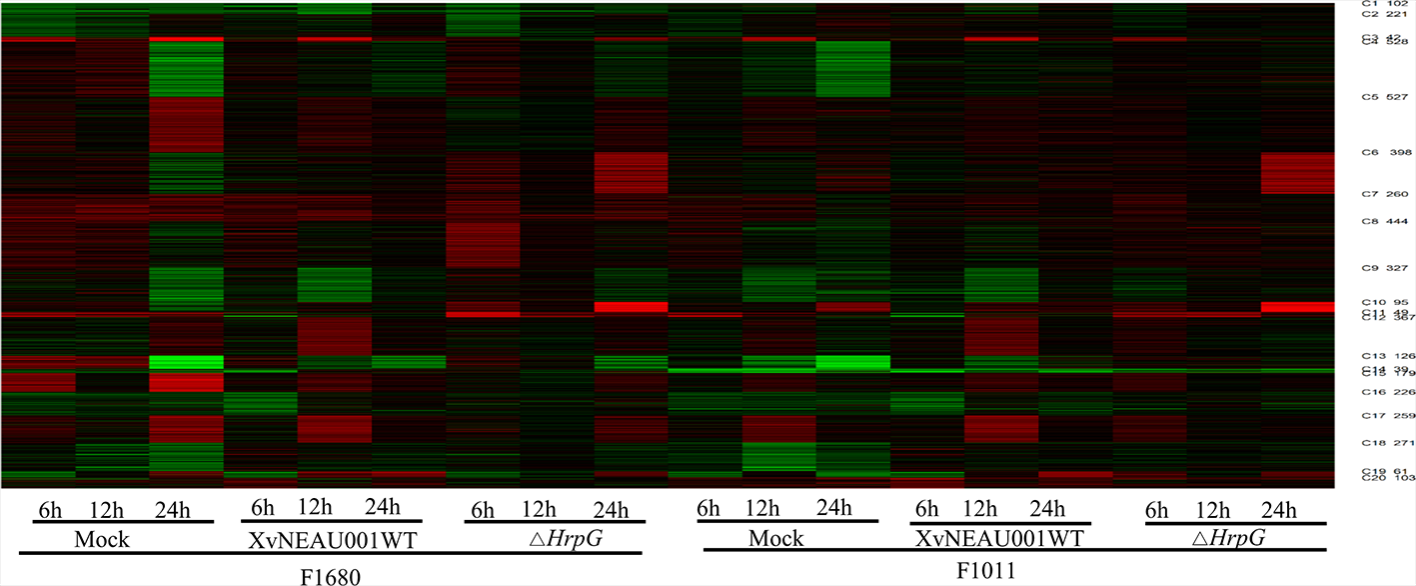
Weighted gene co-expression network analysis (WGCNA) based on the gene expression patterns of Sunong14, F1011, and F1680. **(A)** Hierarchical cluster tree with co-expression modules identified by the WGCNA. Each leaf in the tree represents one gene. The major tree branches constitute 40 modules labeled with different colors. **(B)** Module–sample association. Each row corresponds to a module labeled with a color as in **(A)**. Modules are distinguished by different colors, which were arbitrarily assigned by the WGCNA package. Each column corresponds to a tissue type. The color of each cell at the row–column intersection indicates the correlation coefficient (R) between the module and the tissue type.

The genes/functional pathways linked to soybean responses to bacterial pathogens and type-III effectors may be identified with a weighted gene co-expression network analysis (WGCNA). Thus, we constructed soybean gene co-expression networks based on our RNA-seq data. A total of 39,783 genes were subjected to a WGCNA. We ultimately identified 40 distinct modules when the soft-thresholding power was set at 30. The number of genes in these modules ranged from 156 (coral2) to 8,028 (black). Additionally, 40 co-expression modules were significantly correlated with at least one time-point (P <0.01; **Figure 4**, **Dataset S3**).

The five modules that were positively and negatively correlated with the susceptible line (F1680) and the resistant line (F1011) from 6 to 24 hpi were darkturquoise, darkmagenta, antiquewhite4, lightsteelblue1, and plum1. We also observed that darkslateblue, darksseagreen4, sienna3, bisque, lightpink4, palevioletred3, mediumorchid, and brown4 were only positively correlated with F1680 and F1011, implying these modules may have important functions related to the soybean–pathogen interaction, especially the genes responsive to HrpG regulated bacterial effectors.

**Figure 5:**
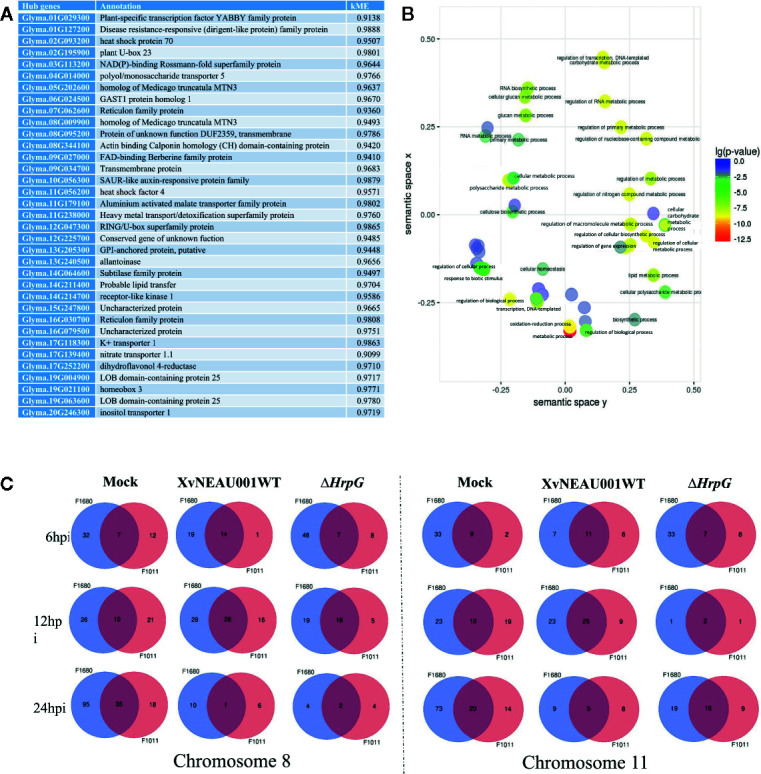
Annotation of genes in significant modules. **(A)** Annotation of candidate hub genes. **(B)** GO annotation of hub genes. **(C)** Two significant hub gene interacting networks in substituted segments of chromosomes 08 and 11.

A focused examination of the substituted segments uncovered 35 hub genes in the substituted region of the wild soybean genome (**Figure 5A**). Most of these genes were grouped in module lightsteelblue1. Additionally, these 35 genes were functionally characterized as involved in transport, establishment of locations, and cell and membrane processes. Specifically, Glyma.19G021100 and Glyma.05G202600 encode proteins belonging to the MtN3 family, which are regulated by bacterial type-III effectors and are likely targets of *Xanthomonas* virulence factors (Streubel et al., 2013; Miao et al., 2018).

To further characterize the genes in the lightsteelblue1 module, a GO enrichment analysis indicated the following GO terms were enriched in this module: regulation of cellular biosynthetic process; regulation of transcription; regulation of primary metabolic process; regulation of nucleobase, nucleoside, nucleotide, and nucleic acid metabolic process; regulation of biosynthetic process; regulation of nitrogen compound metabolic process; regulation of RNA metabolic process; regulation of transcription, DNA-dependent; regulation of macromolecule biosynthetic process; regulation of cellular metabolic process; regulation of gene expression; and regulation of macromolecule metabolic process (**Figure 5B**). These results implied that the genes in the lightsteelblue1 module are largely responsible for regulating metabolic processes.

**Figure 6:**
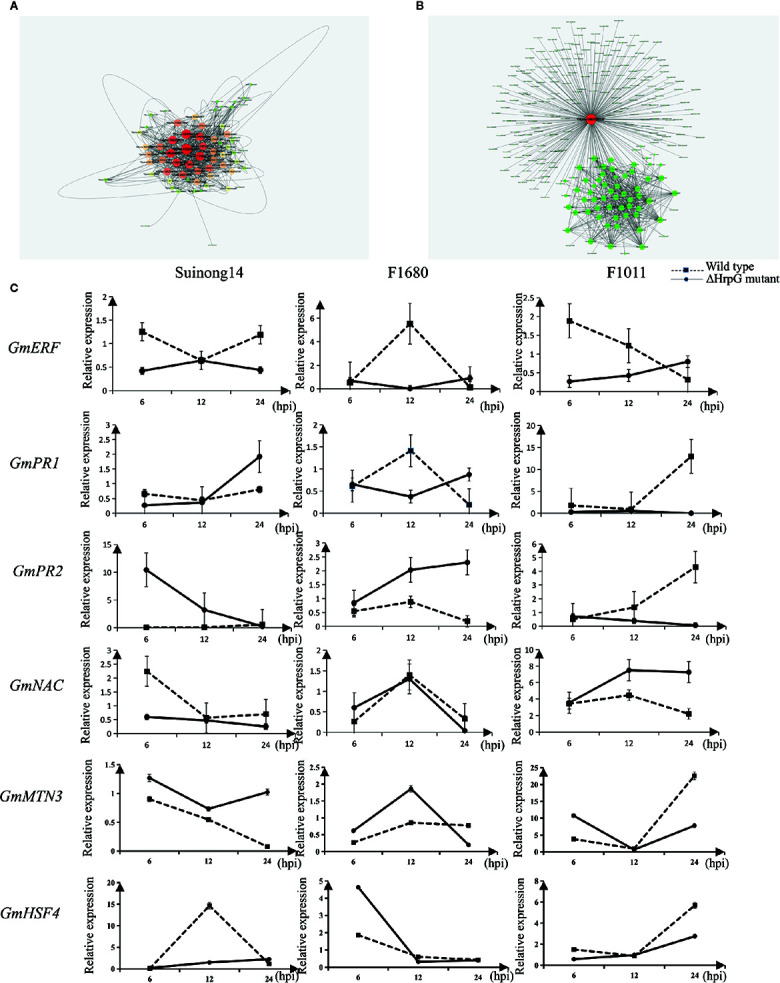
Identification of DEGs on chromosomes 08 and 11. **(A**, **B)** Analysis of Glyma.08G00900.1 and Glyma.11G056200.1, which are two hub genes in a gene interaction network. **(C)** Analysis of candidate gene expression levels in a qRT-PCR assay.

A quantitative real-time polymerase chain reaction (qRT-PCR) assay was completed to validate the RNA-seq results for six genes whose expression differed by more than 3.0-fold between F1011 and F1680 inoculated with XvNEAU001WT and XvNEAU001Δ*hrpG* (**Figure 6C**). These six genes are involved in defense mechanisms [i.e., Glyma.08G009900 (MTN3), Glyma.11G056200 (HSF4), Glyma.15G062500 (PR1), Glyma.03G132700 (PR2), Glyma.11G036400 (ERF), and Glyma.16G019400 (NAC)]. The qRT-PCR results were consistent with the gene expression patterns detected by RNA-seq.

### Characterization of the Hub Genes in Substituted Segments With QTLs Underlying *Xanthomonas* Resistance

Both F1011 and F1680 had no more than one substituted segment. To further narrow down the interesting fragment and candidate genes, we identified QTLs in the RIL population. Our data combined with the reported QTLs revealed several hub genes in the region overlapping the QTL region and substituted segment.

We identified 15 conditional QTLs, 18 QTLs induced by the *hrpG* mutant, and 13 QTLs induced by the wild-type *Xanthomonas*. These QTLs were distributed on chromosomes 01, 02, 04, 05, 06, 07, 08, 10, 11, 12, 15, 16, 17, 18, 19, and 20. One QTL induced by the *hrpG* mutant was located in a chromosome 08 region (**Table S2**) with one substituted segment in F1011, but not in F1680. A more detailed analysis of the genes in this region uncovered Glyma.08G00900, Glyma.08G0095200, and Glyma.08G00900. Additionally, Glyma.08G00900, which encodes MTN3, was localized to a QTL region induced by the *hrpG* mutant, implying this gene contributes to the type-III effector-triggered signaling in *Xanthomonas*. One QTL induced by the *hrpG* mutant and a conditional QTL were co-localized on chromosome 11 within a region with a substituted segment in F1680, but not in F1011. Moreover, Glyma.11G056200, Glyma.11G179100, and Glyma.11G238000 were detected in this region. Glyma.11G056200 encodes heat shock factor 4 (HSF4). In *Arabidopsis thaliana*, HSF4 is involved in pathogen stress signaling mediated by the electrophilic oxylipin PPA1. A mutation to HSF4 can increase the susceptibility of the mutant to bacterial pathogens (Mueller et al., 2008; Sham et al., 2015). To decrease the number of candidate genes, we compared F1011 and F1680 regarding the genes in the substituted segments on chromosomes 08 and 11. Our results implied that these genes were significantly differentially expressed between F1011 and 1680 (**Figure 5C**, **Table S2**). Furthermore, Glyma.08G00900.1 and Glyma.11G056200.1 are two hub genes that are included in the network of interacting genes (**Figures 6A, B**, **Dataset S6**).

### Expression and Haplotype Analysis of Hub Genes

**Figure 7:**
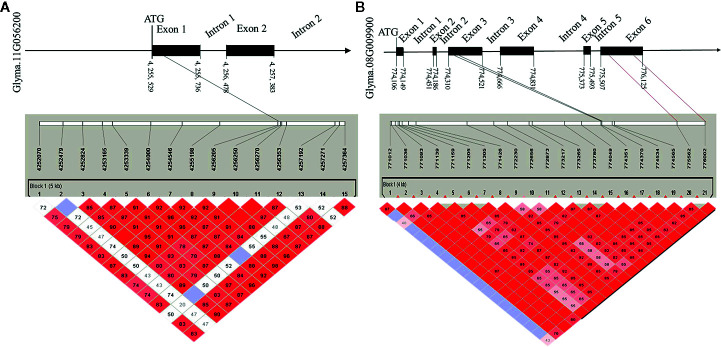
Candidate gene structure in 200 CSSL lines. **(A)** the SNP (single nucleotide polymorphisms) of Glyma. 11G056200 distribution in promoter, exon and intron region. **(B)** the SNP of Glyma. 08G009900 distribution in promoter, exon and intron region.

To determine the haplotypes associated with the 35 identified hub genes, all hub genes were analyzed in 300 soybean core germplasm from the northeastern part of China. We also examined whether these haplotypes exist in the substituted segments of F1011 and F1680. By aligning the DNA sequences of 100 CSSLs, we determined that Glyma.11G056200 is associated with 25 haplotypes. Haplotypes 2 and 23 are two distinct differences between F1068 and F1011. These haplotypes were localized to the exon regions of Glyma.11G056200 (**Figure 7**).

## Discussion

Bacterial leaf pustule is widespread in many soybean-growing regions in northeastern China, with the number of *Xanthomonas*-infected soybean fields steadily increasing. In this study, XvNEAU001WT was isolated from diseased leaves. Interestingly, *Pseudomonas* strains were also isolated from the same leaf materials, possibly there are coincidentally inhabited sites with symptoms.

Our sequence alignments indicated that XvNEAU001WT is highly similar to *X. vasicola* LMG, but not *X. axonopodis* pv. *glycines*, implying the latter is not the only *Xanthomonas* species that causes BLP. Previous study had identified that *X. vasicola* belong to the Cluster I, which is different from included *X. albilineans*, *X. hyacinthi*, *X. sacchari*, *X. theicola* and *X. translucens* (Goncalves and Rosato, 2002). *X. vasicola* had been identified as the pathogen to sugarcane plants (Coutinho et al., 2015), here the XvNEAU001 isolated from the soybean broaden the host arrange of *X. vasicola.*

Our RNA-seq analysis confirmed that the expression of soybean genes is influenced by *Xanthomonas* and the derived *hrpG* mutant. We focused on the genes in the substituted segments. The F1011 and F1680 CSSLs are sib-lines derived from the same parental cultivar, Suinong14. The Glyma.11G056200 (HSF4) gene, but not Glyma.08G009900 (MTN3), was localized to the substituted segments. The *MTN3* gene was first identified as *MtN3* (*Medicago truncatula Nodulin3*), which is involved in *Rhizobium*-induced nodule formation (Gamas et al., 1996). Members of the *MTN3* gene family were subsequently identified in mice, humans, and sea squirts as well as in several plant species, including petunia, rice, and *A. thaliana* (Miao et al., 2018). In rice, specific *X. oryzae* pv. *oryzae* strains appear to use MTN3 to facilitate infections (Yuan et al., 2014). Additionally, MTN3 may contribute to the trafficking of COPT1 and COPT5 to the membrane, wherein the stable COPT1–COPT5 complex mediates the copper influx in rice cells (Yuan et al., 2014). Moreover, GmHSF4 is similar to the *A. thaliana* heat shock promoter elements, which have been identified as one of the main elicitors of plant defense responses to pathogens (Libault et al., 2007). A previous study on pepper proved that HSP family members can interact with the *Xanthomonas* type-III effector AvrBsT to trigger plant cell death and immunity (Kim & Mudgett, 2019). In *A. thaliana*, AtHSP4 is critical for responses to oomycetes, and is suppressed by an oomycete effector to enable infections (Song et al., 2015). Therefore, HSPs can regulate the expression of many genes.

In this study, the expression levels of genes encoding MTN3 and HSF increased after induced by the HrpG mutant, implying that HrpG regulated pathogenic genes may be involved in the MTN3 signaling pathway. Although HrpG is a known regulator of *hrp* expression, there is currently no evidence confirming the ability of HrpG to recognize and bind to the promoter region of the genes it regulates (Ficarra et al., 2015). We speculate that there may genes regulated by HrpG have interact with MTN3. Some secreted effectors of *Xanthomonas* might help regulate *MTN3* expression and sugar transport. In rice, a transcription activator-like (TAL) effector, TAL5, reportedly can induce the expression of *OsSWEET14*, which can increase the susceptibility of rice to *X. oryzae* pv. *oryzae* (Streubel et al., 2013). Earlier investigations revealed that the expression of *SWEET* genes can be induced by bacterial and fungal/pathogens, indicating that SWEET transport is pivotal for pathogens and symbionts (Denancé et al., 2014; Patil et al., 2015). In the current study, a gene structure analysis detected a SNP in the exon, promoter, and intron regions of Glyma.08G009900 and Glyma.11G056200 between F1011 and F1680 (**Figure 7**). This finding suggests that Glyma.08G009900 and Glyma.11G056200 are candidate genes underlying the diversity in the pathogen resistance of F1011 and F1680. Furthermore, future studies should examine the roles of these genes regarding the relationship between soybean and pathogen type-III effectors.

## Materials and Methods

### *Xanthomonas* Isolation and Identification

Fresh leaves exhibiting BLP symptoms were isolated from field-grown soybean plants. The collected leaves were sterilized with 1:1,000 HgCl_2_, after which they were immersed in alcohol and subsequently thoroughly rinsed with sterile water to remove the HgCl_2_ (Fett, 1984). The leaf tissue was then crushed in a tube of sterile water with several sterile metal beads, and then used to inoculate solid King’s medium B lacking antibiotics. Individual clones were isolated for an analysis of the 16S rDNA (Hauben et al., 1997).

### *Xanthomonas* Genome Sequencing and Construction of the *hrpG* Mutant

An individual XvNEAU001WT clone was cultured in liquid King’s medium B until the optical density (600 nm) reached 0.6. The bacteria were harvested to isolate genomic DNA for sequencing by Novogene (http://www.novogene.com/). Specifically, the genome sequencing was completed with the Illumina HiSeq™ 2000 system (sequencing depth of about 100×). All generated reads were assembled with the standard settings of the SOAPdonovo2 assembler software (http://soap.genomics.org.cn/soapdenovo.html) (Luo et al., 2012). Coding sequences were predicted with the GeneMarkS software (http://topaz.gatech.edu/) (Besemer et al., 2001).

To generate the *hrpG* deletion mutant, the sequence from 306 bp upstream to 641 bp downstream of the *hrpG* coding region was ligated into the pKMS1 vector at the *Eco*RI site. Tri-parental mating was used to generate the *hrpG* mutant as previously described (Zou et al., 2011). Details regarding all strains, plasmids, and primers are provided in **Table S1**.

### Plant Materials and Inoculation

Ten soybean germplasm (Suinong14, Hefeng25, Heinong37, Hefeng35, Dongnong42, Sunong10, Dongyinxiaolidou, Dongnong50, Suinong8, and Hefeng1) from Heilongjiang were selected to assess the pathogenicity of XvNEAU001WT. Two CSSLs (F1680 and F1011), their recurrent parent (Suinong14), and an advanced generation RIL population with 143 lines were analyzed (Qi et al., 2014; Xin et al., 2016). Soybean seeds were surface sterilized with chlorine gas as previously described (Zhang et al., 2018). Plants were grown in a greenhouse to evaluate their resistance at the seedling stage. An atomizer was used for the high-pressure inoculation of the upper surface of the first trifoliate leaves with the XvNEAU001WT and the derived *hrpG* mutant culture at an optical density (600 nm) of 0.5 (1 × 10^9^ cells ml^−1^), respectively (Park et al., 2008). The plant inoculations were completed with three replicates, each comprising three leaves. After inoculating the RILs with the *hrpG* mutant and XvNEAU001WT, the disease symptoms were evaluated based on the number of lesions (approximately 0.2 cm^2^) for a subsequent QTL analysis. The phenotype difference between Suinong14, F1680 and F1011 was analyzed by multiple comparison test, which was inoculated with XvNEAU001WT and the derived *hrpG* mutant, respectively.

A population comprising 143 RILs derived from a cross between Charleston (female) and Dongnong594 (male) was used to construct a high-density genetic map based on SLAF sequencing technology, involving 5,308 specific-length amplified fragment markers covering 20 linkage groups, with a total length of 2,655.68 cM (Qi et al., 2014).

### RNA Isolation and Sequencing

Soybean leaf samples (**Figures 1A, B**, **Dataset S1**) were collected from Suinong14, F1680, and F1011 plants at 6, 12, and 24 hpi with the *hrpG* mutant and the parental strain for an RNA-seq analysis, which was completed with three biological replicates. Total RNA was extracted from the collected leaves with TRIzol reagent (Invitrogen). First-strand cDNA was prepared from 1 µg total RNA with the PrimeScript™ RT Reagent Kit (Takara Biomedical Technology, Beijing). Sequencing libraries were constructed with the Illumina TruSeq kit (Illumina Inc., San Diego, CA, USA). The Agilent 2100 and NanoDrop instruments were used to determine the integrity and purity of the total RNA, after which mRNA sequencing was performed to determine the forward or reverse orientation of the DNA strands to more accurately count the number of transcripts and determine the gene structures on the Illumina HiSeq 4000 PE 150 system (Prashant et al., 2013). The sequencing was completed by Novogene (http://www.novogene.com/).

### Analysis of the Transcriptome and Whole-Genome Resequencing Data

We analyzed the RNA-seq datasets with a modified version of a previously published protocol (Liu et al., 2012). The quality of the raw transcriptome data was controlled with the FastQC Toolkit (Hedin et al., 2012). The raw RNA-seq datasets were aligned to the *G. max* Wm82.a2.v1 (https://phytozome.jgi.doe.gov) genome and annotated with HISAT2 (Kim et al., 2015). Read numbers for each gene were determined with HTseq-count (Anders et al., 2015) based on a GTF-formatted file generated by Cufflinks and Cuffcompare (Cole et al., 2012). The fragments per kilobase of exon per million fragments mapped (FPKM) (Mortazavi et al., 2008) values for the assembled transcripts were calculated and normalized with Cuffcompare, Cuffdiff, and the DESeq2 (Love et al., 2014) R package, with global normalization parameters.

Gene expression levels were normalized and differences in expression were analyzed based on the negative binomial distribution of the DESeq2 R package (Love et al., 2014). The criteria for identifying DEGs were normalized expression fold-change >2 and *P*-value <0.01. The DEGs were aligned to the annotated soybean genome. The DEG expression patterns were classified by *K*-means clustering (Saeed et al., 2003). A GO enrichment analysis was performed with AgriGO (http://bioinfo.cau.edu.cn/agriGO), with a *P*-value <0.01. Additionally, the Kyoto Encyclopedia of Genes and Genomes pathway enrichment analysis was performed with the clusterProfiler R package. The WGCNA (Langfelder et al., 2008) was completed with the default settings of the WGCNA R package, with the following exceptions: power was 8, TOMType was unassigned, minModuleSize was 100, and mergeCutHeight was 0.25. The Cytoscape software was used for visualizing networks. The hub genes were mined from the co-expression network. The txt output was managed with our own python script, and all images were developed with the corresponding R package.

### RNA Isolation and qRT-PCR Analyses of Candidate Genes

To verify whether the candidate genes were involved in nodulation, total RNA was extracted with a total RNA extraction reagent (Transgene Biotech Co, Beijing, China). First-strand cDNA was synthesized by reverse transcription with the HiScript^®^ II First-strand cDNA Synthesis Kit (Vazyme Biotech, Nanjing, China). The qRT-PCR assay was completed with the Roche LightCycler 480 quantitative PCR instrument. The qRT-PCR primers were designed based on the locus information in Phytozome, and *GmUKN1* (Glyma.12G020500) was used as a reference gene for normalizing the target gene expression levels (Hu et al., 2009). The qRT-PCR program was as follows: pre-denaturation at 95°C for 30 s and then 40 cycles of release and an annealing temperature of 58°C for 10 s. The qRT-PCR assay was completed with three biological replicates, each comprising three technical replicates, to increase the accuracy of the data.

### QTL Localization

To identify the QTLs underlying the BLP phenotype, a previously described composite interval mapping method (Jiang and Zeng, 1995) involving WinQTL Cartographer (Wang et al., 2012) was used. Specifically, five control markers and a 10-cM window size were used as well as a walk speed of 0.5 cM and the forward regression method.

The difference between the phenotypic value induced by the parental strain (XvNEAU001WT) and the phenotypic value induced by the XvNEAU001Δ*hrpG* mutant was used to determine the locations of the conditional QTLs (Zhu, 1995).

## Data Availability Statement

The datasets generated for this study can be found in NCBI Bioproject ID PRJNA579398.

## Author Contributions

JZou, ZZ, QK, YS, JinW, and RZ contributed equally to this work. All authors contributed to the article and approved the submitted version.

## Conflict of Interest

The authors declare that the research was conducted in the absence of any commercial or financial relationships that could be construed as a potential conflict of interest.

## Supplementary Material

Figure S1 The genomic schematic of substituted segments in F1011 and F1680.

Table S1 Annotation of hub genes in the substituted segments (kME > 0.95, r > 0.85).

Table S2 Localization of QTLs in an RIL population following the inoculations with XAvNEAU001 and the derived hrpG mutant.

Table S3 GO analysis result.

Dataset S1 Annotation of samples.

Dataset S2 Expression patterns of gene clusters.

Dataset S3 Genetic information based on the WGCNA.

Dataset S4 Differentially expressed genes in substituted segments of chromosomes 08 and 11.

Dataset S5 Gene locations in substituted segment blocks.

Dataset S6 Genes included in the Glyma.08G00900.1 and Glyma.11G056200.1 network.
