# Supplementary material for: Responses of Soybean Genes in the Substituted Segments of Segment Substitution Lines Following a Xanthomonas Infection
Source: Front Plant Sci. 2020 Jul 2;11:972. doi: 10.3389/fpls.2020.00972 (PMC7351525; doi:10.3389/fpls.2020.00972)
Supplement: Figure S1 — The genomic schematic of substituted segments in F1011 and F1680. [file DataSheet_1.zip › New folder/Figure S 12019-12-21.pptx]

## Slide 1
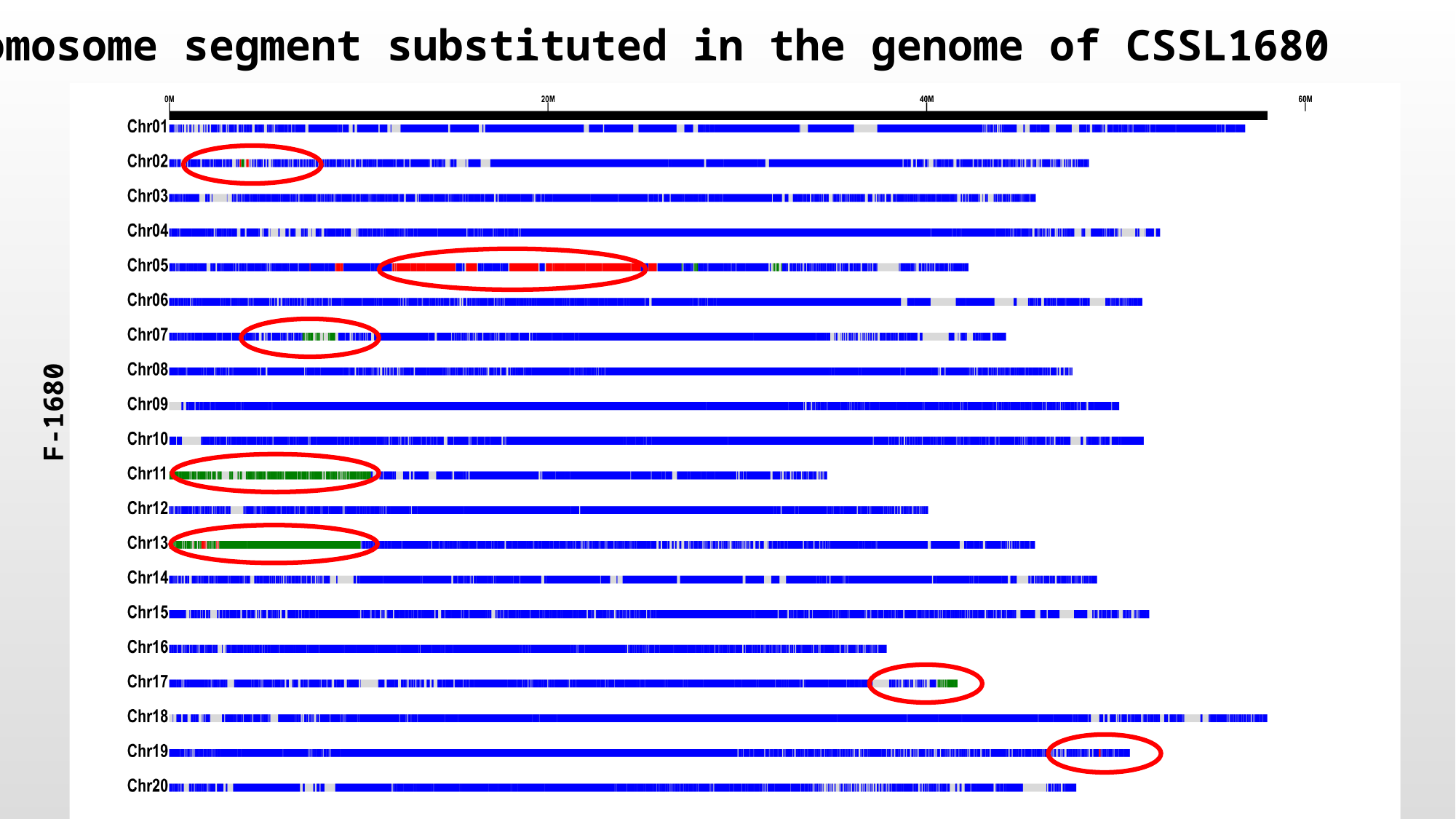

Chromosome segment substituted in the genome of CSSL1680
F-1680

## Slide 2
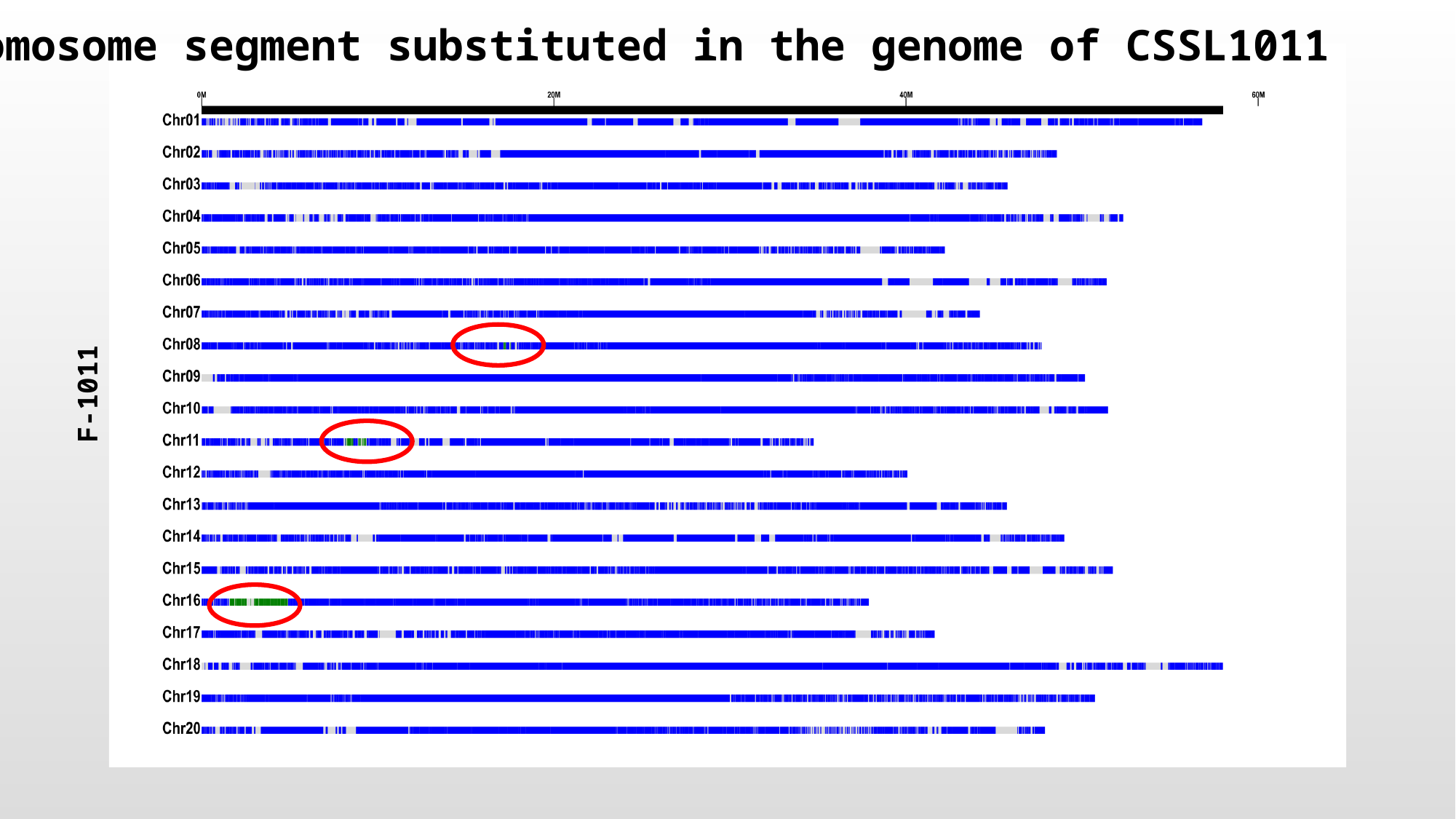

Chromosome segment substituted in the genome of CSSL1011
F-1011
