# Supplementary material for: Responses of Soybean Genes in the Substituted Segments of Segment Substitution Lines Following a Xanthomonas Infection
Source: Front Plant Sci. 2020 Jul 2;11:972. doi: 10.3389/fpls.2020.00972 (PMC7351525; doi:10.3389/fpls.2020.00972)
Supplement: Figure S1 — The genomic schematic of substituted segments in F1011 and F1680. [file DataSheet_1.zip › New folder/Table S1 and Table S2.docx]

Table S1 Annotation of hub genes in substituted segment (kME>0.95, r>0.85)

| **Strain** | **Relevant characteristics** | **Reference** |
| --- | --- | --- |
| ***Escherichia coli*** |  |  |
| DH5α | supE44 lacY169 ( 80lacZM15) hsdR17 recA1 endA1 gyrA96 thi-1 relA1 | Mason *et al*. 1989 |
| ***Xanthomonas*** |  |  |
| XvNEAU001 | A wild type *Xanthomonas* strain from the soybean leaf with BLP phenotype in the field | This study |
| XvNEAU001△HrpG | *XvNEAU001* derived mutant, the coding sequence of HrpG was deleted completely from the genome of XvNEAU001 | This study |
| Help strain | *E.coli* contain the Tra^+^ helper plasmid pRK2013 for mobilization (Kan+) | Figurski and Helinski, 1979 |
| **Plasmid** |  |  |
| pGWC | Entry clone vector,Cm^r^ | Chen *et al*. 2006 |
| pKMS1 | Kan+ , 6 400 bp, pUC18 polylinker, *mob, oriV, sacB* | *Zou et al., 2011* |
| **Primers** |  |  |
| ΔhrpG-up-F | TCCCGGGGCAGCTGACAGT | This study |
| ΔhrpG-up-R | GGAATTCGCAAGCGCACCG | This study |
| ΔhrpG-down-F | AGAATTCCGATCCGGCGTTG | This study |
| ΔhrpG-down-R | CCTCTAGAGTCGACTCGCCGGT | This study |
| 16s-27F | AGA GTT TGA TCC TGG CTC AG | This study |
| 16s-1492R  M13-20 | TAC GGC TAC CTT GTT ACG ACT T  GTT GTA AAA CGA CGG CCA G | This study |
| M13-26 | CAG GAA ACA GCT ATG AC | This study |
| Glyma.11G056200q-F | GTTGTTGTTGTTGTCTAAAGCAAA | This study |
| Glyma.11G056200q-R | GACTCTTATATAATACGGAACCCA | This study |
| Glyma.08G009900q-F | ATTTAATGAAGGGATACATGTGCA | This study |
| Glyma.08G009900q-R | CACAGAAAACAAAACAGAGGTTAC | This study |
| PR1-F | aactatgctccccctggcaactatattg | This study |
| PR1-R | tctgaagtggtagcttctacatcgaaacaa | This study |
| PR2-F | tgaaataagggccacgagtccaaatg | This study |
| PR2-R | atggtacatgcagacttcaagaatgcagat | This study |
| ERFq-F | CCAACCACACGCTGCAAATCG | This study |
| ERFq-R | CCTCTGTAGTGCTTCTTCTCTTCCG | This study |
| NACq-F | GGAGAACCACCACAACACCAACA | This study |
| NACq-R | CCCTTCATTGTCCGTGCCCATC | This study |

Table S2 QTL location result in RIL population by inoculate with XAvNEAU001 and the derived HrpG mutant.

|  | HrpG mutant | | XavNEAU001 | | | Conditional QTL | | |
| --- | --- | --- | --- | --- | --- | --- | --- | --- |
| Chromosome | Position | LR | Chromosome | Position | LR | Chromosome | Position | LR |
| 1 | 42.390351 | 117.5948 | 1 | 45.039748 | 4.302249 | 2 | 21.195175 | 14.65952 |
| 1 | 26.493969 | 108.9903 | 2 | 26.493969 | 5.895675 | 2 | 71.533717 | 7.648443 |
| 4 | 29.143366 | 110.1376 | 2 | 76.832511 | 5.736332 | 2 | 68.88432 | 7.648443 |
| 4 | 66.234923 | 157.1755 | 4 | 55.637336 | 7.250087 | 7 | 50.338542 | 12.49246 |
| 8 | 66.234923 | 102.1067 | 5 | 31.792763 | 10.91497 | 12 | 7.948191 | 16.1892 |
| 10 | 23.844572 | 118.1684 | 6 | 47.689145 | 7.329758 | 11 | 15.896382 | 10.26166 |
| 11 | 18.545779 | 126.7729 | 10 | 5.298794 | 13.78313 | 10 | 29.143366 | 7.71218 |
| 15 | 42.390351 | 114.7266 | 10 | 23.844572 | 13.22543 | 12 | 55.637336 | 8.477024 |
| 15 | 23.844572 | 102.6803 | 12 | 2.649397 | 21.98927 | 19 | 31.792763 | 15.10568 |
| 16 | 31.792763 | 122.7575 | 17 | 76.832511 | 6.055017 | 19 | 10.597588 | 11.15398 |
| 17 | 10.597588 | 163.4855 | 19 | 7.948191 | 13.3051 | 20 | 47.689145 | 10.38914 |
| 17 | 13.246985 | 165.2064 | 19 | 29.143366 | 12.42872 | 16 | 10.597588 | 11.15398 |
| 17 | 13.246985 | 163.4855 | 20 | 10.597588 | 7.090744 | 17 | 82.131305 | 10.45287 |
| 18 | 95.37829 | 153.1601 |  |  |  | 19 | 26.493969 | 13.89467 |
| 19 | 47.689145 | 152.0128 |  |  |  | 16 | 26.493969 | 9.560554 |
| 20 | 39.740954 | 138.2456 |  |  |  |  |  |  |
| 18 | 60.93613 | 128.4938 |  |  |  |  |  |  |
| 18 | 31.792763 | 113.5794 |  |  |  |  |  |  |
